# The first evidence of Asian-like CPV-2b in Slovakia in a vaccinated dog with an acute fatal course of parvovirus infection: a case report

**DOI:** 10.1007/s11259-024-10492-z

**Published:** 2024-08-09

**Authors:** Andrea Pelegrinová, Patrícia Petroušková, Ľuboš Korytár, Anna Ondrejková, Monika Drážovská, Boris Vojtek, Jana Mojžišová, Marián Prokeš, Maroš Kostičák, Ľubica Zákutná, Michal Dolník, René Mandelík

**Affiliations:** 1 Department of Epizootiology, Parasitology and Protection of One Health, University of Veterinary Medicine and Pharmacy in Košice, Komenského 73, Košice, 041 81 Slovakia; 2 Clinic of Ruminants, University of Veterinary Medicine and Pharmacy in Košice, Komenského 73, Košice, 041 81 Slovakia

**Keywords:** CPV-2, Infection, Dogs, Phylogenetic analysis, Slovakia

## Abstract

This study provides a comprehensive description of the clinical course of a fatal parvovirus infection in a vaccinated dachshund puppy, along with the first identification of a new CPV-2 variant in Slovakia, elucidated through molecular amino acid analysis of the VP2 gene. The dog exhibited clinical signs such as apathy, vomiting, and bloody diarrhea. After confirming CPV-2 infection with a commercial snap test, intensive therapy was initiated. The dog succumbed within 48 h of admission. A rectal swab sample was collected, CPV-2 was examined using the PCR method, and sequenced. The virus detected in the patient was related to strains of CPV-2c of Asian origin and unrelated to European CPV-2b strains. The sequence had genetic signatures typical of Asian strains (VP2: 5Gly, 267Tyr, 324Ile, 370Arg, and 440Thr). Phylogenetic analysis classified this strain as similar to Asian strains of CPV-2c. It is believed to be derived from an Asian strain similar to CPV-2c that acquired the 426Asp mutation. With this finding, we present the first evidence of an Asian-like CPV-2b strain in the territory of Slovakia.

**Supplementary Information:**

The online version contains supplementary material available at 10.1007/s11259-024-10492-z.

## Introduction

Canine parvovirus type 2 (CPV-2) is the cause of highly contagious acute enteritis associated with high morbidity and mortality, with a dubious prognosis survivability in untreated dogs. The first case of canine parvovirus infection was reported in 1978 (Appel et al. 1979). The virus underwent genetic modifications in the capsid protein VP2, giving rise to new variants CPV-2a, CPV-2b, and CPV-2c (Miranda and Thompson 2016). While severe clinical signs primarily occur in puppies and young dogs up to the age of 6 months, adult dogs with weakened immunity can also be potentially affected (Duarte et al. 2013). The main clinical signs of parvovirus infection are hemorrhagic diarrhea and vomiting, in rare cases myocarditis is observed (Tavora et al. 2008). Non-specific clinical signs such as depression, lethargy, and pyrexia are often observed (Nandi and Kumar 2010). Despite the use of effective vaccines, CPV-2 infection has been reported even in vaccinated dogs (Tuteja et al. 2022). The main causes of vaccine failure include interference with maternal antibodies and incorrect vaccination timing (Altman et al. 2017). For these reasons, CPV-2 remains a global problem responsible for severe and deadly infections in puppies (Pratelli et al. 2001). CPV-2 variants circulate on all continents to varying extents. Most current studies have analyzed amino acid substitutions in the VP2 gene sequence (Ala5Gly, Phe267Tyr, Ser297Ala, Tyr324Ile, Gln370Arg, Thr440Ala, and Ala514Ser). These unique mutation sites are associated with virulence and immune escape of the virus (Mira et al. 2018). Several studies confirm the spread and introduction of CPV-2c strains of Asian origin from Asia to Europe via imported dogs, confirming the role of dog transportation in global virus spread (Mira et al. 2018, 2019). These strains are characterized by a specific set of mutations in structural and non-structural proteins and are phylogenetically related to each other (Balboni et al. 2021; Canuti et al. 2022). Systematic epizootiological surveys are needed to evaluate the circulation, development, and spread of new CPV-2 variants in different geographical areas (Schirò et al. 2022). This study provides a detailed description of the clinical course of a fatal parvovirus infection in a vaccinated dachshund puppy. It also reports the first discovery of a new CPV-2 variant in Slovakia, identified through molecular amino acid analysis of the VP2 gene.

## Patient history

A 10-month-old male dachshund (date of birth: 9th October 2021), weighing 5.9 kg upon admission to the hospital, presented as the patient (Supplementary Figs. 1, 2). The dog received his primary vaccinations on November 23, 2021, with the DHPPi vaccine. It was revaccinated on December 14, 2021, with the vaccine DHPPi + L vaccine, and a second revaccination was done on January 5, 2022, with the DHPPiLR vaccine. The dog was admitted to the University Veterinary Hospital of the University of Veterinary Medicine and Pharmacy in Košice on 7th August 2022 (Sunday) with reported symptoms of apathy, vomiting, and diarrhea lasting for 2 days. The patient’s rectal temperature upon admission was 38.3 °C. Based on the history and clinical signs, suspicion of parvovirus disease was raised, and a chromatographic immunological test was conducted on a rectal swab sample to qualitatively detect the antigen of canine parvovirus (CPV/Ag), canine coronavirus (CCV/Ag), and *Giardia* (Giardia/Ag) (Antigen Rapid CPV/ CCV/ Giardia Ag; Bionote, Hwaseong-si, Gyeonggi-do, Korea). The immunochromatographic test was positive for CPV/Ag, while CCV/Ag and Giardia/Ag were negative. The dog was hospitalized for two days until its death. The death occurred on August 9, 2022, due to total organ failure.

## Materials and methods

The suspicion of disease was based on the patient’s medical history and clinical examination. A detailed description of the clinical examination is provided in Supplementary Material. The diagnosis was established based on serological and molecular methods supplemented by virus cultivation and isolation on the cell line.

### Antigen rapid testing

The dog was tested on August 7, 2022, using the Antigen Rapid CPV/ CCV/ Giardia Ag (Bionote, Hwaseong-si, Gyeonggi-do, Korea) test directly at the first contact clinic. Two rectal swabs were used as samples for this test. The test was performed according to the manufacturer´s instructions (Supplementary Fig. 3).

### Hematological and biochemical blood examination

As part of a comprehensive examination, the dog’s blood profile and selected biochemical indicators were monitored in peripheral blood taken from the cephalic antebrachial vein on August 7, 2022. Hematological examination was performed using the IDEXX ProCyte Dx instrument, and biochemical examinations were carried out on the IDEXX Catalyst One instrument.

### Bacteriological and mycological cultivation

On August 7, 2022, a rectal swab was taken due to bloody diarrhea in the dog. The rectal swab sample was sent to the Central Veterinary Laboratory, Likavka-Ružomberok, of Unilabs Slovensko, s.r.o. Aerobic cultivation was performed in the laboratory.

### Serological examination for IgM and IgG antibody detection

A serological examination for the detection of IgG and IgM antibodies against CPV-2 was performed on August 10, 2022. Serological tests were performed to obtain protective antibody titers (IgG, IgM) to provide information on the efficacy of previous vaccination and the presence of an acute infection. The serum sample was sent to the Central Veterinary Laboratory, Unilabs Slovensko, s. r.o. in Likavka-Ružomberok. Interpretation of serological examination results: a titer of 1:30 indicates undetected antibodies, titers of 1:90 and 1:270 indicate detected antibodies, and a titer of 1:810 indicates a high antibody titer.

### Molecular detection of CPV-2 DNA and sequencing

The extraction of viral DNA from the rectal swab was performed using the DNeasy^®^ Blood & Tissue kit (Qiagen, Hilden, Germany) following the manufacturer’s instructions (www.qiagen.com/HB-2061, accessed on September 8, 2023).

The extracted DNA from the rectal swab sample was confirmed by PCR, targeting a 573 bp VP2 fragment (nucleotides 9-581) of CPV-2 using a universal set of primers for all CPV-2 types (Table 1). As a positive control, laboratory-confirmed CPV-2 was used. PCR-amplified product stained with GelRed^®^ Nucleic Acid Stain (Merck Millipore, Massachusetts, USA) was subjected to gel electrophoresis in a 1.0% agarose gel in Borax buffer, visualized by UV transilluminator (MiniBIS Pro imaging system, Marcoussis, France), and captured by GelCapture™ software for the expected product size of 573 bp.

**Table 1:** Primers used for CPV-2 detection and full-length VP2 sequencing in this study

Primer name	Sequence (5´ − 3´)	Position^a^	Size (bp)	Reference
*CPV-2 detection*				
degCPV-VP2-F	TGATGGAGSAGTWCAACCAGA	2791–2811	573	this study
CPV-VP2-R	TCAGATCTCATAGCTGCTGGA	3343–3363		Li et al. 2022
*CPV-2 sequencing*				
VP2-F^b^	AGAGACAATCTTGCACCAAT	2768–2787	554	Hu et al. 2020
CPV-VP2-INT-R1	CTATCTAATGCAACCATCAATG	3300–3321		this study
CPV-VP2-INT-F1	GTTGCATTTAGTTAGTTTTGAACA	3190–3213	541	this study
CPV-VP2-INT-R2	ACCACGTCTTTTATCTTGTTG	3710–3730		this study
CPV-VP2-INT-F2	GATTGTAAACCATGTAGACTAACA	3590–3613	563	this study
CPV-VP2-INT-R3	GCAGTTAAAGGACCATAAGTA	4132–4152		this study
CPV-VP2-INF-F3	GAAGATATCCAGAAGGAGATTGG	4005–4027	539	this study
VP2-R^c^	ATGTTAATATAATTTTCTAGGTGCT	4519–4543		Hu et al. 2020

^a^Primer positions are based on the nucleotide position in the canine parvovirus 2 complete genome (NCBI Reference Sequence: NC_001539)

^b^Please note that this primer comprises the amplification also of the 3´end part of the VP1 gene (18 bp)

^c^Please note that this primer comprises the amplification also of the initial part (3 bp) of the 3´ untranslated region (3´UTR)

The CPV-2-confirmed sample (Supplementary Fig. 4A) was then subjected to the amplification of the complete VP2 sequence (1,755 bp) by three individual PCR reactions using specific pairs of primers (Table 1). Positive amplicons (Supplementary Fig. 4B) of expected size (554 bp, 541 bp, 563 bp, and 539 bp) were purified using the Wizard^®^ SV Gel and PCR Clean-Up System according to the manufacturer’s instructions (Promega, Wisconsin, USA) and sequenced by the Sanger method using the primer pairs used for amplification of each fragment. Detailed information on the primers is shown in Table 1. Please note that all primers were designed by alignment of full-length VP2 gene sequences of CPV-2 types (CPV-2a, b, and c) available in the GenBank database on October 15, 2023, to produce overlapping fragments. The final VP2 sequence (1,755 bp) was assembled according to the overlapping strategy and aligned using a Geneious v9.1.8 program (Biomatters, San Diego, CA, USA) including the reference gene for the canine parvovirus VP2 protein (GenBank ID: M38245) to ensure the alignments were within the correct framework.

### Analysis of amino acid residues of the VP2 protein

The VP2 nucleotide sequence was translated into the amino acid (aa) sequence to determine the antigenic variant of CPV-2 (2a, 2b, or 2c) based on the aa in position 426 and substitutions of selected aa (5, 267, 297, 324, 370, and 440) along the VP2 protein. The analysis was conducted using the Geneious v9.1.8 program (Biomatters, San Diego, CA, USA). Substitutions at sites 5, 267, 324, 370, and 440 were compared with amino acid variations of European, Asian, and American CPV-2 reference strains obtained from GenBank (accessed on January 19, 2024).

To model the position of aa substitution in the isolate from this study, the Phyre online platform (http://www.sbg.bio.ic.ac.uk/phyre2/html/page.cgi?id=index, accessed on January 16, 2024) (Kelley et al. 2015) was used. The amino acids were represented using the EzMol v2.1 molecular modeling web software (http://www.sbg.bio.ic.ac.uk/ezmol/, accessed on January 16, 2024) (Reynolds et al. 2018).

### Phylogenetic analysis

The phylogenetic relationships of the analyzed CPV-2 strain with other CPV-2 strains were elucidated using MEGA v11 software (Kelley et al. 2015). For a more comprehensive evaluation, 61 sequences of the VP2 gene were downloaded from GenBank as reference sequences based on genotype and geographic origin (https://www.ncbi.nlm.nih.gov/genbank/, Supplementary Table 4). Sequences were edited and aligned using a ClustalW method implemented in MEGA v11 software. The phylogenetic tree was constructed by a maximum likelihood statistical method using the Tamura-3 parameter model (T92) with discrete Gamma distribution (five rate categories) (G) and invariant sites (I). To access the confidence level of the branching pattern, a bootstrap analysis with 1,000 bootstrap replicates was performed. Bootstrap values > 50 were indicated at the corresponding node.

### Cell culture and virus isolation

Madin-Darby Canine Kidney (MDCK) cell line was used to isolate CPV from a stool sample. Briefly, MDCK cell line was grown to confluence in Minimal Essential Medium (MEM) (Sigma-Aldrich, Darmstadt, Germany) supplemented with 10% fetal bovine serum (FBS; Sigma) and 1% antibiotic antimycotic solution (10,000 U/mL penicillin, 10 mg/mL streptomycin, and 25 µg/mL amphotericin B; Sigma-Aldrich, Darmstadt, Germany) at 37 °C with 5% CO_2_ with 95% humidity.

The virus isolation was performed as described by (Hirayama et al. 2005). The virus supernatants were screened for the presence of the virus by PCR using the primer pair used for CPV-2 detection (Table 1).

### Pathological examination

The autopsy of the cadaver was performed at the Veterinary and Food Institute in Košice on August 10, 2022.

## Results

### Antigen rapid testing

The dog was tested positive for CPV/Ag and negative for CCV/Ag and *Giardia*/Ag (Supplementary Fig. 3).

###  Hematological and biochemical blood examination

The blood analysis revealed leukopenia, lymphocytopenia, reduced MCV, and decreased RETIC-HGB (Supplementary Table 1). Based on the biochemical examination, elevated ALT values of 305 U/L (RI: 10–125) were detected. The serum showed hyponatremia of 140 mmol/L (RI: 144–160) and hypochloremia of 108 (RI: 109–122). Other biochemical parameters were within the normal range (Supplementary Table 2).

### Bacteriological and mycological cultivation

No findings of enteropathogenic *Escherichia coli* were observed. Aerobic cultivation revealed a normal intestinal microflora. Mycological cultivation did not yield any findings. Cultivation for *Campylobacter* spp. was also negative.

### Serological examination for IgM and IgG antibody detection

The serum sample contained IgM antibodies against canine parvovirus at a titer of 1:90. In serological examination for IgG antibodies against canine parvovirus, no canine parvovirus antibodies were detected (1:30 titer) (Supplementary Table 3).

### Molecular detection of CPV-2 DNA and sequencing

The VP2 sequence (1,755 bp) was submitted to the GenBank database using the BankIt submission portal (https://www.ncbi.nlm.nih.gov/WebSub/index.cgi) and is openly available in GenBank under accession number OR825360. The assembled nucleotide sequence was submitted to BLASTn (https://blast.ncbi.nlm.nih.gov/Blast.cgi; accessed on June 21, 2024) to search for related sequences in public databases. The comparison of the 1,755 bp long sequence with parvovirus VP2 sequence data reported in GenBank showed the highest nucleotide identities with strains belonging to two types of CPV-2, i.e., CPV-2b strains collected recently in Italy (99.83%−99.77%) and Hungary (99.83%), and CPV-2c strains found in samples collected in China (99.60% – 99.49%), Nigeria (99.77%–99.54%), Thailand (99.54%), Italy (99.54%), Indonesia (99.54%), Gabon (99.54%), Ethiopia (99.54%), Vietnam (99.54%), Canada (99.54%), Korea (99.54%), and India (99.54%) (Supplementary Table 5). Interestingly, all these CPV-2c strains belonged to the Asian CPV-2c clade (see below). The identity with original CPV-2 and CPV-2a was not found.

### Analysis of amino acid residues of the VP2 protein

The amino acid analysis antigenically characterized the Slovakia/Kosice/47/2022 isolate as CPV-2b based on the presence of Asp (D) at residue 426 of the VP2 protein. The isolate was also classified as “new CPV-2a/CPV-2b” due to S297A substitution. A comparative analysis of the Slovakia/Kosice/47/2022 isolate with CPV-2 reference strains at selected aa positions (5, 267, 324, 370, and 440) revealed the pattern of aa substitutions characteristic of Asian CPV-2c strains (5G, 267Y, 324I, 370R, and 440T) (Table 2) suggesting it is more closely related to Asian CPV-2c strains than to CPV-2b strains. The CPV-2b isolate from this study shares the same aa characteristics with other recently described CPV-2b isolates from Italy (OR463607 and ON677437) and Hungary (ON733252) (Table 2), referred to as Asian-like CPV-2b. This allows the classification of the Slovakia/Kosice/47/2022 isolate as a member of the Asian-like CPV-2b strains group.

**Table 2:** Amino acid variations in the VP2 sequence of the CPV-2b isolate analyzed in this study and reference CPV-2 strains

Type	Country	Year	Strain/isolate	Accession number	Amino acid position of VP2 gene					
					5	267	324	370	426	440
CPV-2	USA^a^	1979	CPV-5.us.79	EU659116	A	F	Y	Q	N	T
CPV-2^a^	Italy^b^	2009	CPV_IZSSI_29451_09	KX434454	A	F	Y	Q	N	A
	India^c^	2013	CPV/915-H	KF366250	A	F	Y	Q	N	T
	Canada^a^	2016	CPV/Raccoon/RC19/BC_2016	MF069443	A	F	Y	Q	N	T
	China^c^	2014	CPV/CN/SD9/2014	KR002802	A	Y	I	Q	N	A
CPV-2b	Portugal^b^	2013	PT077/13	KR559895	A	F	Y	Q	D	T
	Japan^c^	2017	9985	LC270891	A	F	Y	Q	D	T
	Ecuador^a^	2012	2b_ME20_ECU2012	KF149985	A	F	Y	Q	D	S
	China^c^	2018	CPV-AHmas3	MT648206	A	Y	I	Q	D	A
CPV-2c	France^b^	2009	202-09	MF177227	A	F	Y	Q	E	T
	Uruguay^a^	2010	UY242	KM457120	A	F	Y	Q	E	T
Asian CPV-2c	China^c^	2022/2023	CPV2/2022/3	OR399577	G	Y	I	R	E	T
	Italy^b^	2022	IZSSI_2022PA17019idC1	OR463608	G	Y	I	R	E	T
	Canada^a^	2018	FM4	OM640098	G	Y	I	R	E	T
Asian-like CPV-2b	Italy^b^	2022	CPV-2b_IZSSI_2022PA2773	ON677437	G	Y	I	R	D	T
	Hungary^b^	2021	FR1/CPV2-2021-HUN	ON733252	G	Y	I	R	D	T
	**Slovakia**^**b**^	**2022**	**Slovakia/Kosice/47/2022**	OR463608	**G**	**Y**	**I**	**R**	**D**	**T**

A – Alanine, D – Aspartic acid, E – Glutamic acid, F – Phenylalanine, G – Glycine, I – Isoleucine, N – Asparagine, Q – Glutamine, R – Arginine, S – Serine, T – Threonine, Y – Tyrosine

^a^American CPV-2 reference strains

^b^European CPV-2 reference strains

^c^Asian CPV-2 reference strains. The sequence analyzed in this study is displayed in bold

### Phylogenetic analysis

**Fig. 1:**
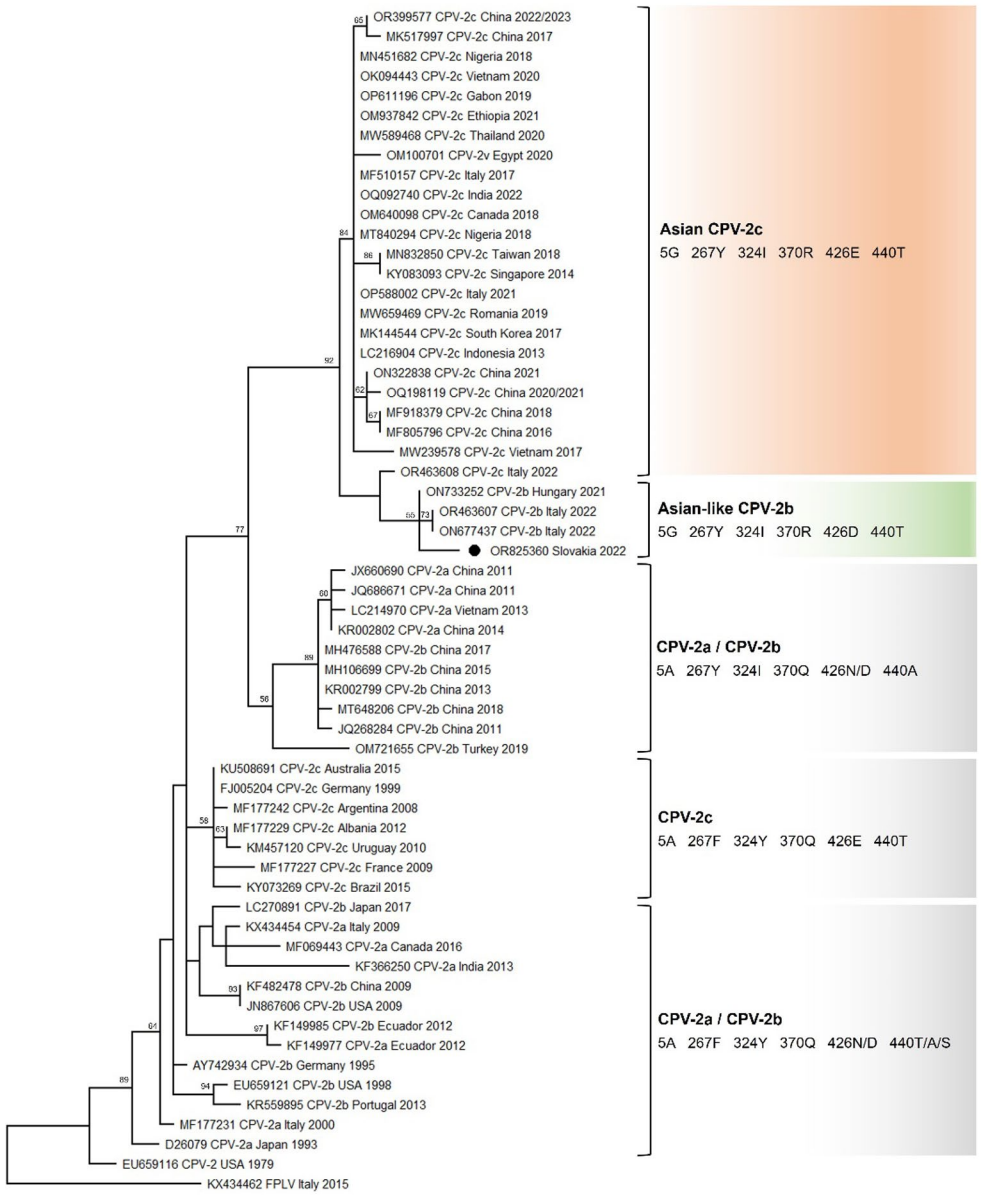
Phylogenetic analysis of the Slovakia/Kosice/47/2022 isolate. Phylogenetic analysis of VP2 nucleotide sequences of canine parvovirus. The phylogenetic tree was constructed based on the complete VP2 gene nucleotide sequences (1,755 bp) of canine parvovirus (CPV) obtained in this study and reference strains in the GenBank database (Supplementary Table 4 ). Feline panleukopenia virus (KX434462) was used as an outgroup. Bootstrap values greater than 50% are indicated on the respective branches. The phylogenetic tree was constructed by MEGA X v11 using the maximum likelihood statistical method and Tamura-3 parameter model (T92) with discrete gamma distribution (five rate categories) (G) and invariant sites (I). Statistical support was provided by bootstrapping with 1.000 replicates. The black circle represents the sequence identified in this study (OR825360). The antigenic variants and selected amino acid residues in positions 5, 267, 324, 370, 426, and 440 are reported

The phylogenetic analysis confirmed that the Slovakia/Kosice/47/2022 isolate belongs to a group (highlighted in green in Fig. 1) corresponding to Asian-like CPV-2b strains collected in Italy in 2022 (OR463607 and ON677437) and Hungary in 2021 (ON733525), indicating a close phylogenetic relationship. Moreover, the phylogenetic tree showed an incorporation of Slovakia/Kosice/47/2022 isolate into one cluster, supported by a high bootstrap value, which groups the Asian CPV-2c (highlighted in red in Fig. 1) characterized by the VP2 amino acid residues 5G, 267Y, 324I, 370R, and 440T. The group of Asian-like CPV-2b strains is closely related to the Italian CPV-2c strain (OR463608) (Fig. 1).

### *Cell culture and virus isolation*

**Fig. 2:**
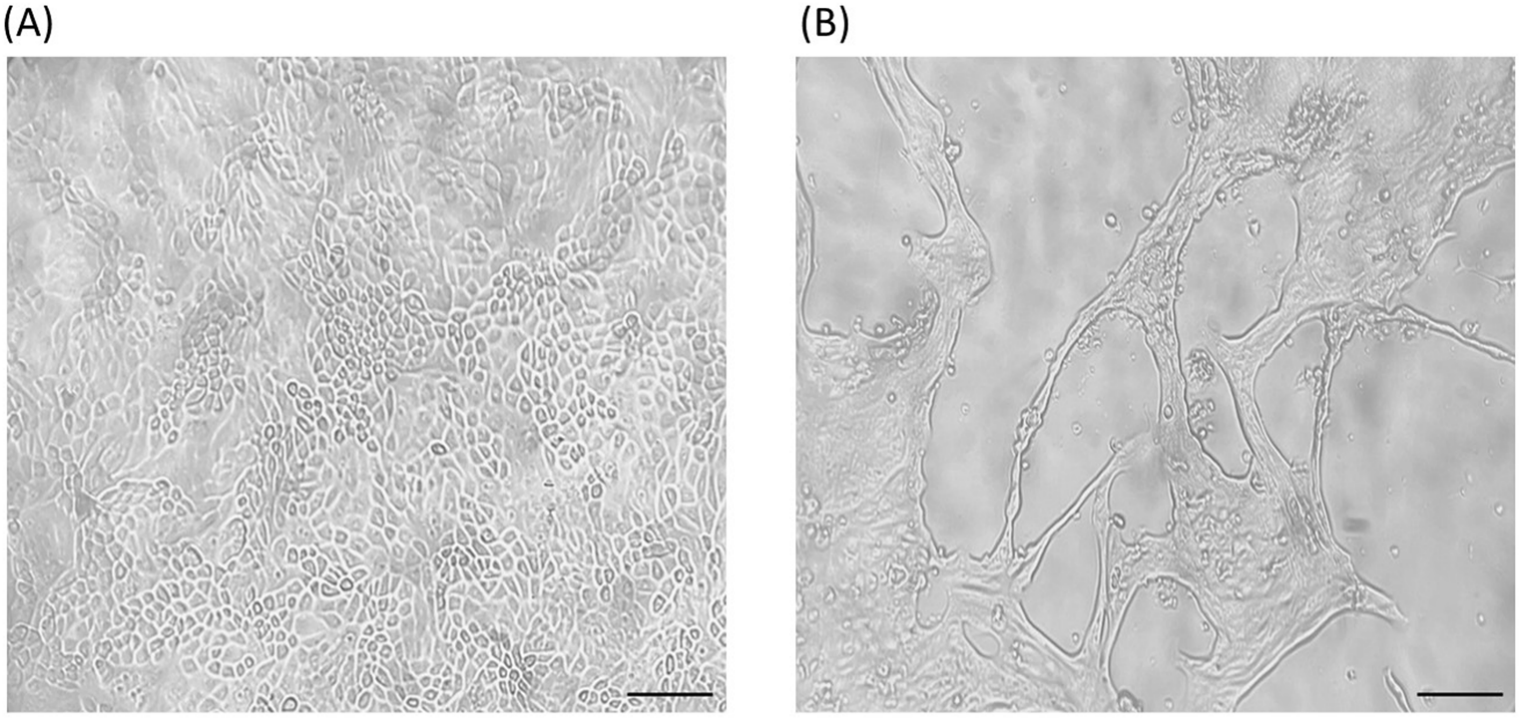
Cytophatic effect of CPV-2b on MDCK cell culture. **A** Uninfected MDCK cells used as a negative control; **B** Cytopathic effect of CPV-2b characterized by forming of plaques and disruption of the monolayer. The cytopathic effect in the MDCK cell line was visible four days post-infection. Scale bar – 50 μm

Typical CPE in the form of plaque-forming cells and partial lysis of the monolayer was observed on 4th day after inoculation at the first passage level. No detectable changes were seen in the negative control sample (exclusion of inoculum incubation) (Fig. 2). The presence of the virus in the harvested culture supernatant was confirmed by the amplification of the 573 bp fragment of the VP2 gene (Supplementary Fig. 4C) using the universal set of primers (Table 1).

###  Pathological examination

The pathological examination revealed reduced skin elasticity, bilateral serous nasal discharge mixed with bile, and anemia of the oral mucosa. The respiratory system was affected by pulmonary parenchymal congestion with extensive hemorrhages, as well as frothy discharge in the trachea, bronchi, and bronchioles mixed with stomach contents (Supplementary Fig. 5). The cardiovascular system showed miliary to petechial hemorrhages on the epicardium and mild right ventricular hypertrophy (Supplementary Fig. 6). Gastrointestinal findings included petechial hemorrhages in the gastric serosa, hemorrhagic enteritis, and intestinal contents mixed with bile and hemolyzed blood (Supplementary Fig. 7). There was also evidence of liver (Supplementary Fig. 8) and kidney hyperemia (Supplementary Fig. 9), along with multiple hemorrhages in the splenic parenchyma (Supplementary Fig. 10). The combination of these lesions led to systemic organ failure and resulted in fatality.

## Discussion and conclusions

In this article, we describe a clinical case of Asian-like origin of CPV-2b in a vaccinated dog.

The diagnosis was established by a rapid chromatographic immunological test for the qualitative detection of canine parvovirus, canine coronavirus, and giardia in the stool (Desai et al. 2020). Suspicions of parvovirus disease were confirmed by a Rapid Test - Antigen Rapid test kit CPV/CCV/Giardia Ag, with a positive result for CPV/Ag from a rectal swab sample (Mazzaferro 2020). White blood cells participate in the body’s defense against infections, their reduced production in the bone marrow occurs as a result of viral infection (Mylonakis et al. 2016). The blood analysis revealed leukopenia, lymphocytopenia, reduced MCV and decreased RETIC-HGB. Bacteriological and mycological cultures yielded a negative finding, likely due to the administration of antibiotics by another veterinarian before the collection of the rectal swab sample. In addition to the microbiological examination, a coprological examination was also performed. The presence of parasites was not detected, and the dog was dewormed by Caniverm tablets (Bioveta^R^) in 3 terms. Vaccination is the best prevention against the onset, spread, and development of clinical CPV-2 infection (Ellis et al. 2022). Primary vaccination and revaccination were carried out within the prescribed period (14–21 days) as recommended by the manufacturer (Bioveta^R^). The interval between the second and third revaccination of the vaccine (Bioveta^R^) was 22 days, and the third revaccination was performed with a one-day delay. IgG and IgM antibody titers are among the most commonly used correlates of protection, also used to determine previous exposure (IgG), detect immunity induced by vaccination (IgG), or infection during the acute phase of inflammation (IgM) (Earle et al. 2021). In the serum sample, IgM antibodies against canine parvovirus were found at a titer of 1:90, confirming an acute CPV-2 infection. No canine parvovirus IgG antibodies were detected.

Polymerase chain reaction (PCR) combined with sequencing is the most commonly used molecular method for pathogen detection (Prasad 2017). The presence of parvovirus infection in the puppy was confirmed using multiple techniques. In addition to the rapid antigen test and conventional PCR test (Supplementary Fig. 4A), the identification of specific mutations in the VP2 protein, namely K80R, K93N, V103A, and D323N, which are characteristic of canine parvoviruses as distinct from feline parvoviruses (Zhou et al. 2017), provided further validation of the diagnosis. As described by Miranda et al., variant CPV-2a typically features asparagine (Asn, N), variant CPV-2b exhibits aspartic acid (Asp, D), and CPV-2c variant is characterized by glutamic acid (Glu, E) (Miranda et al. 2017). Based on this classification scheme, the isolate identified in our study (Slovakia/Kosice/47/2022) was categorized as a CPV-2b variant. An additional amino acid substitution occurring at position 297 of the VP2 protein (Ser to Ala), detected for the first time in 1990, has been identified within our sequence. This mutation serves as a distinctive genetic marker associated with emerging variants of new CPV-2a and new CPV-2b, highlighting evolutionary dynamics within the virus (Ohshima et al. 2008). However, in contrast to European CPV-2b strains, the CPV-2b isolate Slovakia/Kosice/47/2022 displays notable amino acid alterations within the VP2 protein, which are typical of Asian CPV-2c strains (A5G, F267Y, Y324I, Q370R) (Table 2). In this regard, a novel cluster of CPV-2b strains with CPV-2c-like genetic signatures, known as Asian-like CPV-2b, has been recently described (Schirò et al. 2022b). CPV-2b isolates retrieved from Hungary in 2021 (ON733252) (Boros et al. 2022) and Italy in 2022 (OR463607 and ON677437) share the same amino acid characteristics at residues 5, 267, 324, 370, and 440 of VP2 protein as were observed in 98.6% of the strains within the Asian CPV-2c clade. Schirò and colleagues proposed a potential origin for this cluster as a result of acquiring the E426D substitution through convergent evolution (Schirò et al. 2022b). This hypothesis can be further supported by the observation that 426 is the frequent mutation site (Voorhees et al. 2019). The CPV-2b isolate from this study, along with Asian-like CPV-2b strains identified in Italy and Hungary, are found to be phylogenetically related to Asian CPV-2c strains within the same clade (Fig. 1). This finding supports that they share a common evolutionary trajectory, possibly originating from CPV-2c strains. Since the Asian CPV-2c strains are already circulating in Europe (Franzo et al. 2023), the European origin of Asian-like CPV-2b strains cannot be ruled out. However, their geographical origin remains unclear. Previous studies reported that the epidemic characteristics of five mutant sites in VP2 protein (A5G, F267Y, Y324I, and Q370R) were associated with CPV-2 antigenicity and host range (Sarabandi and Pourtaghi 2023). Among these mutations, A5G and Q370R have been identified as exclusive mutations to CPV-2c (Hao et al. 2022). These mutations have been also detected in isolates belonging to the Asian-like CPV-2b, including the isolate analyzed in this study. The amino acid residue 5 is located in at the N-terminus within a crucial antigenic region of the VP2 protein (Langeveld et al. 1993). Therefore, A5G mutation, resulting from a C → G transversion, may have an impact on the virus antigenicity and immunogenicity. The A5G mutation often appeared together with F267Y, Y324I, and Q370R mutations (Wang et al. 2016; Hao et al. 2022). The Q370R mutation was first identified in CPV-2a strains detected from giant pandas in China (Guo et al. 2013) and subsequently became the predominant mutation in CPV-2c strains (Hao et al. 2022). Since the residue 370 is located around the protein-binding pocket on a loop of VP2 (Supplementary Fig. 11), the Q370R mutation could potentially trigger a conformational shift or contribute to altered virus-receptor binding affinity by modulating interactions between the VP2 protein and host DNA (Guo et al. 2013; Hao et al. 2022). Both the 5G and Q370R mutations have been documented in CPV strains from different geographic regions, including Hungary (Boros et al. 2022), Italy (Mira et al. 2019), Romania (Balboni et al. 2021b), Nigeria (Ogbu et al. 2020; Tion et al. 2021), Egypt (Ndiana et al. 2022), China (Hao et al. 2020; Li et al. 2022), Thailand (Charoenkul et al. 2019; Inthong et al. 2020), and Vietnam (Doan et al. 2021), indicating their widespread distribution and global dissemination. As mentioned, these mutations can have significant implications for host range and VP2-binding capacity (Hao et al. 2022). However, the effectiveness of vaccines used for parvovirus prophylaxis against the 5G and 370R mutants remains uncertain. Amino acid residues 267 and 324 are situated in a GH loop, a high antigenic motif within the VP2 protein (Supplementary Fig. 11) essential for viral entry via transferrin receptor-binding region (Tsao et al. 1991; Zhou et al. 2017). These residues are believed to play a pivotal role in the evolutionary process leading to the emergence of new CPV variants (Nandi et al. 2019). Mutation F267Y and Y324I are thought to be Asian strains characteristic. Specifically, F267Y mutation was first reported in 2002 in the Chinese CPV-2c isolates and has become predominant since 2014 (Zhou et al. 2017), and Y324I mutation has been prevalent in Asian countries (Yoon et al. 2009; Zhang et al. 2010; Phromnoi et al. 2010; Soma et al. 2013; Mukhopadhyay et al. 2014). Recent studies have reported the presence of these mutations in CPV strains from various geographic regions beyond Asia, including Italy (Mira et al. 2019), Colombia (Giraldo-Ramirez et al. 2020), Uruguay (Maya et al. 2013), Brazil (de Oliveira et al. 2019), and Australia (Kwan et al. 2021). All these findings indicate that these mutations are no longer strictly associated with the Asian region, highlighting the ongoing evolutionary progression of CPV-2.

The CPV-2b isolate confirmed in this study was cultured on the MDCK cell line to monitor the CPE. The CPE induced by CPV-2b isolate was observed on the 4th day after infection by plaque-forming and partial lysis of the MDCK monolayer. These changes are indicative of viral-induced cell damage and help in identifying the presence and pathogenicity of CPV-2b isolate. The presence of CPV-2b in cell culture was also confirmed by PCR (Supplementary Fig. 4C).

Pathological diagnosis of CPV-2 consists of the identification of intestinal necrotizing and hemorrhagic lesions, secondary villous collapse, and crypt dilatation with necrotic debris (Sherding 2006). Pathological examination revealed pulmonary parenchymal congestion with hemorrhages, petechial hemorrhages on the epicardium and stomach, hemorrhagic enteritis, hemorrhages in the splenic parenchyma, liver and spleen hyperemia.

In conclusion, the Slovakia/Kosice/47/2022 isolate identified in this study is antigenically classified as CPV-2b, but phylogenetically is a part of the Asian CPV-2c clade with which it shared several mutations in VP2 capsid protein. Considering the mentioned factors, these mutations may have an impact on the effectiveness of current vaccines and the virus’s ability to evade the canine immune system. This could also lead to the potential inadequate immune response and explain why, despite being vaccinated, a puppy from this study succumbed to the parvovirus infection in a short time.

## Supplementary Information

Additional file 1.

Additional file 2.

Additional file 3.

## Data Availability

No datasets were generated or analysed during the current study.
