# Supplementary material for: The first evidence of Asian-like CPV-2b in Slovakia in a vaccinated dog with an acute fatal course of parvovirus infection: a case report
Source: Vet Res Commun. 2024 Aug 9;48(5):3253–62. doi: 10.1007/s11259-024-10492-z (PMC11442606; doi:10.1007/s11259-024-10492-z)
Supplement: Supplementary file 1 — Additional file 1. [file 11259_2024_10492_MOESM1_ESM.pdf]

Title

The first evidence of Asian CPV-2c-like CPV-2b in Slovakia in a vaccinated dog with an acute fatal course of parvoviral infection: a case report

Journal

Veterinary Research Communications

Authors

Andrea Pelegrinová [1], Patrícia Petroušková [1*], Ľuboš Korytár [1], Anna Ondrejková [1], Monika Drážovská [1], Boris Vojtek [1], Jana Mojžišová [1], Marián Prokeš [1], Maroš Kostičák [1], Ľubica Zákutná [1], Michal Dolník [2], René Mandelík [1*]

The affiliations of the authors

[1] Department of Epizootiology, Parasitology and Protection of One Health, University of Veterinary Medicine and Pharmacy in Košice, Komenského 73, 041 81 Košice, Slovakia

[2] Clinic of Ruminants, University of Veterinary Medicine and Pharmacy in Košice, Komenského 73, 041 81 Košice, Slovakia

[*] Corresponding authors. E-mail addresses: rene.mandelik@uvlf.sk; patricia.petrouskova@uvlf.sk

Supplementary Material 1 Clinical examination

The clinical examination was conducted according to the clinical examination protocol.

1. **Behavior and Reactions:** The dog is apathetic but responsive to the external environment.
2. **Vital Signs:** Upon admission, the rectal temperature was 38.48 ºC (reference value "RH": <39.2 ºC), heart rate was 80/min. (RH: 80-160/min.), the pulse at the femoral artery was strong and regular, and the respiratory rate was 28/min. (RH: 15-30/min.). On August 9, 2022, the rectal temperature was 36.7 ºC, subsequently varying throughout the day: 38.3 ºC, 38.4 ºC, 37.0 ºC, 37.1 ºC, 38.2 ºC, 38.1 ºC, 37.7 ºC.
3. **Body Condition Score (BCS):** 2/5, the body condition was less than good upon admission. Ribs were easily palpable, visible thoracic vertebrae processes, sunken abdomen. The dog's weight upon admission to hospitalization on August 7, 2022, was 5.9 kg. During hospitalization, the BCS worsened, and at the time of death on August 9, 2022, the BCS was 1/5, indicating a very poor body condition, with ribs, vertebral processes, pelvic bones, and all bony prominences visible from a distance. A significant loss of muscle mass was noted, and the dog weighed 5.3 kg on the day of death.
4. **Mucous Membranes, CRT, and Hydration**: Pink mucous membranes, moist, prolonged capillary refill time ("CRT") of more than 2 seconds, borderline hydration. Skin turgor returned to normal within 3 seconds. On August 8, 2023, the conjunctiva was brick-red and septic, and the mucous membranes were anemic.
5. **Skin and Coat:** The coat was free of ectoparasites, without pruritus, and the skin appeared normal with no signs of inflammation.
6. **Head:** The facial region showed no pathological changes, was symmetrical, without swelling, and no head tilt.
7. **Eyes (right and left):** The size of the eyeballs was equal, and their position was symmetrical. The eyelids were free of swelling, pink conjunctiva with serous discharge from both eyes. The cornea was transparent, and the anterior chamber, iris, and lens showed no pathological findings on both sides.
8. **External Ear Canals:** Pink and without abnormal discharge or cerumen on both sides.
9. **Nostrils:** Symmetrical, no pathological discharge detected from the left and right nostrils, and no swelling in the anatomical area.
10. **Oral Cavity:** Pink oral mucous membranes, permanent teeth without observed dental calculus. The hard and soft palate, tongue, and pharyngeal area showed no pathological findings.
11. **Neck:** The trachea was non-irritating upon palpation, and coughing was not observed. The thyroid gland was not enlarged.
12. **Cardiovascular System:** Symmetrical chest, heart rate of 80/min. with regular, palpable heartbeats synchronous with the pulse in the femoral artery on both sides. Cardiac auscultation revealed no audible heart murmurs bilaterally.
13. **Respiratory Tract:** A symmetrical chest, costal-abdominal breathing, and a respiratory rate of 28/min. on admission. Vesicular breathing was present, and auscultation of the pulmonary fields showed no abnormal sounds bilaterally. In the terminal stage of the disease, rapid, shallow breathing with audible rales was observed bilaterally.

14. **Abdomen:** The abdomen was palpable, slightly painful initially, and later became significantly painful throughout its extent (cranial, middle, and caudal abdomen). Fluid was present upon ballottement, and the stool was brown with blood admixture.
15. **Urogenital System:** An intact male with scrotum free of skin changes. Prepuce and glans penis were without inflammatory changes or discharge. Testicles were symmetrical, both descended into the scrotum, and urination was spontaneous, with macroscopically yellow urine.
16. **Central and Peripheral Nervous System:** Consciousness: apathetic. Body posture: normal. Gait: overall weakness due to illness. Cranial nerves: no pathological findings. Reflexes of thoracic and pelvic limbs: no pathological findings. Other spinal reflexes: no pathological findings. Sensitivity: preserved surface and deep sensitivity. Postural reactions: no pathological findings.
17. **Musculoskeletal System:** Upon palpation of the spine and joints of thoracic and pelvic limbs, no swelling, pain, deformities, or crepitus were noted. Upon admission, muscle atrophy was not observed. In the terminal stage of the disease, there was significant loss of muscle mass.
18. **Superficial Lymph Nodes**: Submandibular and popliteal lymph nodes were palpable, symmetrical, non-enlarged, firm, freely movable, and non-painful. Suprascapular lymph nodes were non-palpable. Assessment of the Presence of Tumors and Hernias: No tumors or hernias were detected.
