# Supplementary material for: The first evidence of Asian-like CPV-2b in Slovakia in a vaccinated dog with an acute fatal course of parvovirus infection: a case report
Source: Vet Res Commun. 2024 Aug 9;48(5):3253–62. doi: 10.1007/s11259-024-10492-z (PMC11442606; doi:10.1007/s11259-024-10492-z)
Supplement: Supplementary file 2 — Additional file 2. [file 11259_2024_10492_MOESM2_ESM.pdf]

**Title**

# The first evidence of Asian-like CPV-2b in Slovakia in a vaccinated dog with an acute fatal course of parvovirus infection: a case report

**Journal**

Veterinary Research Communications

**Authors**

Andrea Pelegrinová[1], Patrícia Petroušková[1*], Ľuboš Korytár[1], Anna Ondrejková[1], Monika Drážovská[1], Boris Vojtek[1], Jana Mojžišová[1], Marián Prokeš[1], Maroš Kostičák[1], Ľubica Zákutná[1], Michal Dolník[2], René Mandelík[1*]

**The affiliations of the authors**

[1] Department of Epizootiology, Parasitology and Protection of One Health, University of Veterinary Medicine and Pharmacy in Košice, Komenského 73, 041 81 Košice, Slovakia

[2] Clinic of Ruminants, University of Veterinary Medicine and Pharmacy in Košice, Komenského 73, 041 81 Košice, Slovakia

[*] Corresponding authors. E-mail addresses: patricia.petrouskova@uvlf.sk; rene.mandelik@uvlf.sk

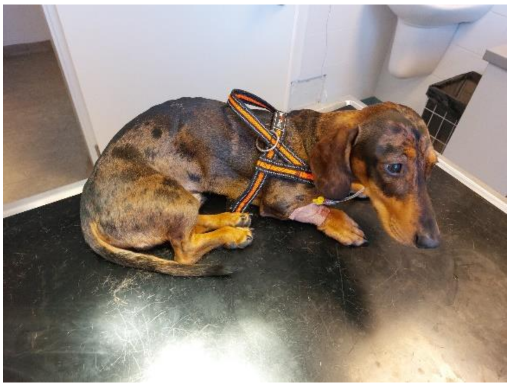

**Supplementary Fig. 1** A ten-month-old male Dachshund with parvovirus disease, lateral view (August 7, 2022)

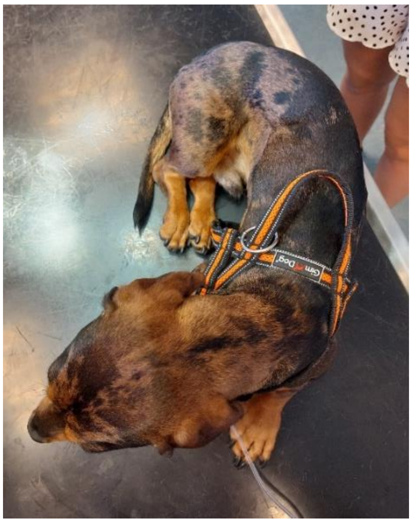

**Supplementary Fig. 2** A ten-month-old male Dachshund with parvovirus disease, dorsal view (August 7, 2022)

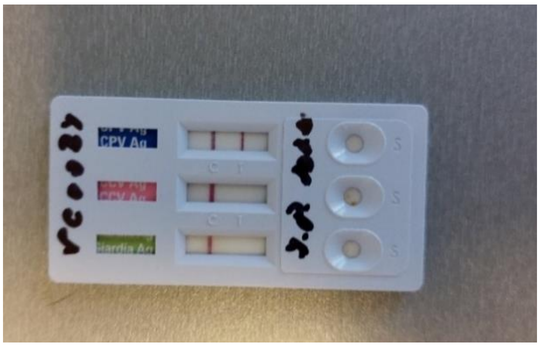


**Supplementary Fig. 3** A positive test for CPV/Ag from rectal swab samples (August 7, 2022)

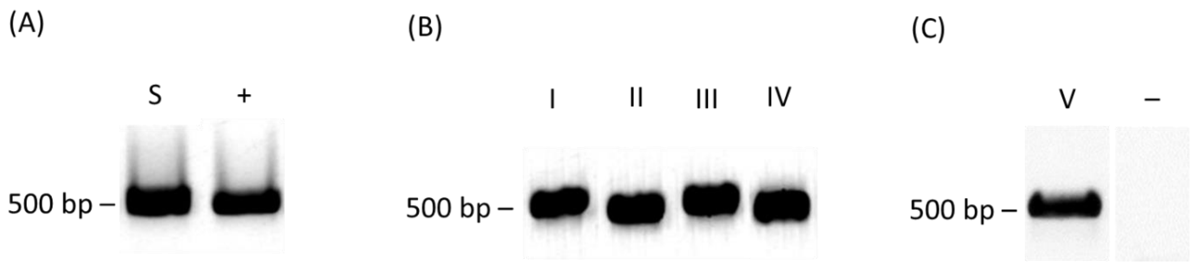


**Supplementary Fig. 4** CPV-2 amplification for partial VP2 fragment and full-length VP2 gene

Visualization of PCR products. (A) Confirmation of parvovirus infection in the sample based on the presence of 573 bp fragment of the VP2 gen. “S” – tested rectal swab sample; “+” – positive control (laboratory-confirmed sample with parvovirus infection). (B) Amplification of overlapping fragments of the VP2 gene for subsequent sequencing. “I” – 554 bp fragment comprising 1 – 536 nucleotides of VP2 gene; “II” – 541 bp fragment that comprises 405 – 945 nucleotides of VP2 gene; “III” – 563 bp fragment comprising 805 – 1367 nucleotides of VP2 gene; “IV” – 539 bp fragment comprising 1216–1755 nucleotides of VP2 gene. (C) Confirmation of the presence of the virus in the harvested MDCK culture supernatant based on the amplification of 573 bp fragment of VP2 gene. “V” – virus presented in the cell culture supernatant on 4$^{th}$ day post-infection; “–“– negative control represented by cell culture supernatant from non-infected cells.

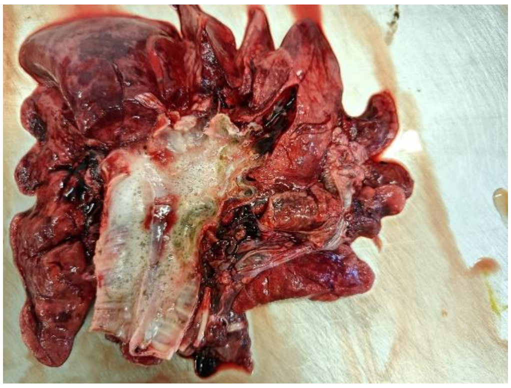

**Supplementary Fig. 5** Foamy discharge in the trachea and bronchi (August 10, 2022)

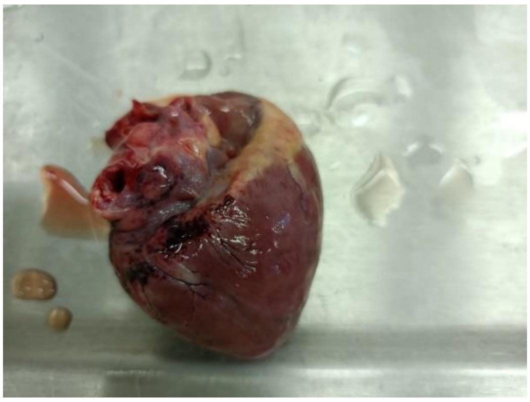

**Supplementary Fig. 6** Petechial hemorrhages on the epicardium (August 10, 2022)

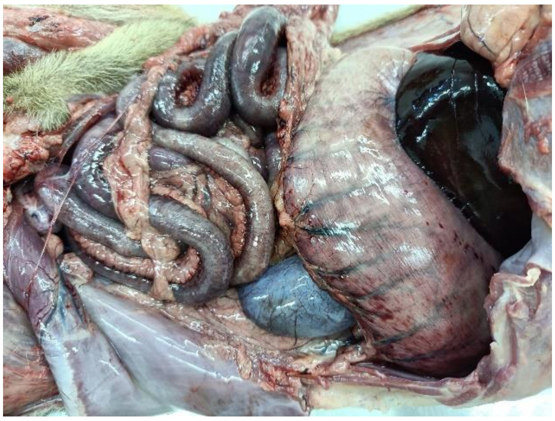

**Supplementary Fig. 7** Hemorrhagic enteritis and petechial hemorrhages on the gastric serosa (August 10, 2022)

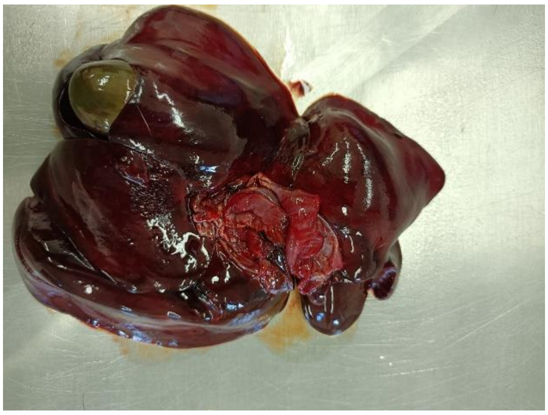

**Supplementary Fig. 8** Hepatic hyperemia (August 10, 2022)

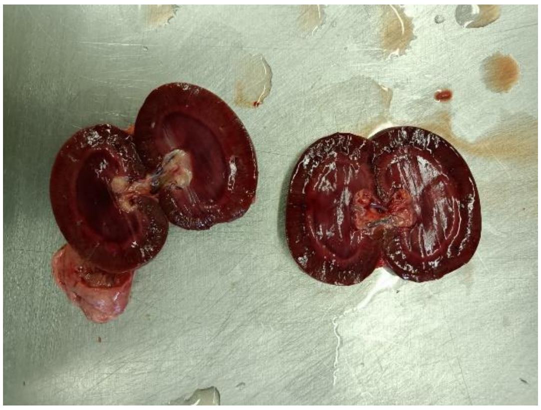

**Supplementary Fig. 9** Renal hyperemia, hemorrhagic inflammation of the kidney cortex and medulla (August 10, 2022)

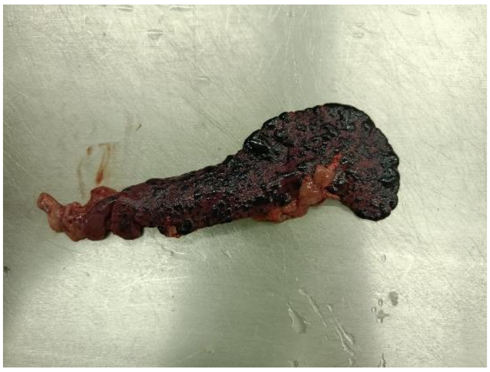

**Supplementary Fig. 10** Multiple hemorrhages in the spleen parenchyma (August 10, 2022)

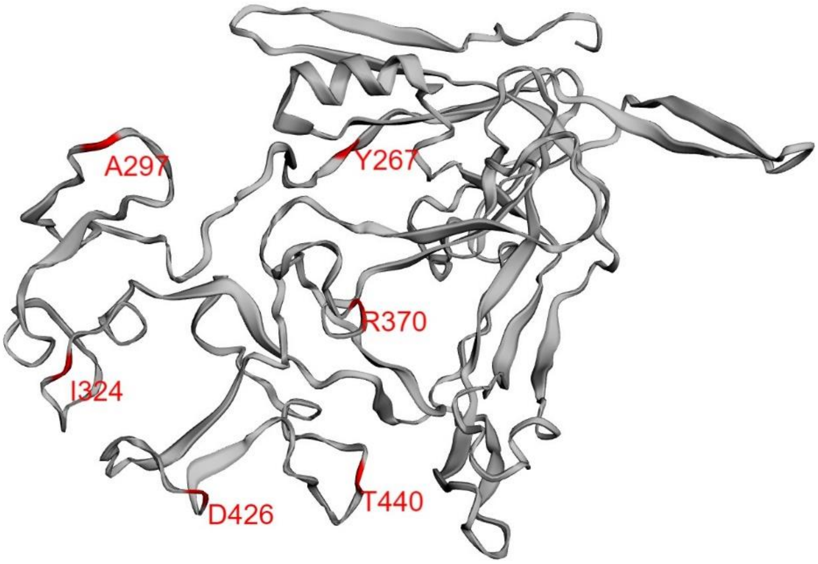


**Supplementary Fig. 11** VP2 protein

Modeling of the VP2 protein structure indicating the key amino acid substitutions in Asian CPV-2c-like CPV-2b variant at sites 267, 297, 324, 370, 426, and 440. For the modeling of amino acid substitutions, the Phire² platform (http://www.sbg.bio.ic.ac.uk/phyre2/html/page.cgi?id=index, accessed on January 16, 2024) (Kelley et al. 2015) was used and visualization was performed by the EzMol v2.1 molecular modeling web software (http://www.sbg.bio.ic.ac.uk/ezmol/, accessed on January 16, 2024) (Kelley et al. 2015).
