# Supplementary material for: The first evidence of Asian-like CPV-2b in Slovakia in a vaccinated dog with an acute fatal course of parvovirus infection: a case report
Source: Vet Res Commun. 2024 Aug 9;48(5):3253–62. doi: 10.1007/s11259-024-10492-z (PMC11442606; doi:10.1007/s11259-024-10492-z)
Supplement: Supplementary file 3 — Additional file 3. [file 11259_2024_10492_MOESM3_ESM.pdf]

Title

The first evidence of Asian-like CPV-2b in Slovakia in a vaccinated dog with an acute fatal course of parvovirus infection: a case report

Authors

Andrea Pelegrinová[1], Patrícia Petroušková[1*], Ľuboš Korytár[1], Anna Ondrejková[1], Monika Drážovská[1], Boris Vojtek[1], Jana Mojžišová[1], Marián Prokeš[1], Maroš Kostičák[1], Ľubica Zákutná[1], Michal Dolník[2], René Mandelík[1*]

The affiliations of the authors

[1] Department of Epizootiology, Parasitology and Protection of One Health, University of Veterinary Medicine and Pharmacy in Košice, Komenského 73, 041 81 Košice, Slovakia

[2] Clinic of Ruminants, University of Veterinary Medicine and Pharmacy in Košice, Komenského 73, 041 81 Košice, Slovakia

[*] Corresponding authors. E-mail addresses: patricia.petrouskova@uvlf.sk; rene.mandelik@uvlf.sk

Supplementary Table 1 Results of blood count and differential blood count

Blood Count	Conclusion	Result	Unit	Reference interval
**RBC**	OK	8.17	10^{12} / L	5.65-8.87
**HCT**	OK	48.9	%	37.3-61.7
**HGB**	OK	18.2	g/dL	13.1-20.5
**MCV**	-	59.9	fL	61.6-73.5
**MCH**	OK	22.3	pg	21.2-25.9
**MCHC**	OK	37.2	g/dL	32.0-37.9
**RDW**	OK	16.8	%	13.6-21.7
**%RETIC**	OK	0.1	%	180.0-500.0
**RETIC**	OK	10.6	K/μL	10.0-110.0
**RETIC-HGB**	-	20.7	pg	22.3-29.6
**WBC**	-	70	%	5.05-16.76
**% NEU**		38.0	%	
**% LYM**		18.9	%	
**% MONO**		8,1	%	
**% BASO**		0.0	%	
**NEU**	OK	3.30	10^9/L	2.95-11.64
**LYM**	-	0.89	10^9/L	1.05-5.10
**MONO**	OK	0.38	10^9/L	0.16-1.12
**EOS**	OK	0.14	10^9/L	0.06-1.23
**BASO**	OK	0.00	10^9/L	0.00-0.10
**PLT**	OK	198	K/μL	148-484
**MPV**	OK	11.7	fL	8.7-13.2
**PDW**	OK	10.0	fL	9.1-19.4
**PCT**	OK	0.23	%	0.14-0.46

RBC–Red Blood Cells, HCT–Hematocrit, HGB–Hemoglobin, MCV–Mean Corpuscular Volume, MCH–Mean Corpuscular Hemoglobin, MCHC–Mean Corpuscular Hemoglobin Concentration, RDW–Red Blood Cell Distribution Width, %RETIC–Percentage of Reticulocytes In The Blood , RETIC–Reticulocytes, RETIC-HGB–Reticulocyte Hemoglobin Ratio, WBC–White Blood Cells, NEU–Neutrophils, LYM–Lymphocytes, MONO–Monocytes, BASO–Basophils, EOS–Eosinophils, PLT–Platelets, MPV– Mean Platelet Volume, PDW–Platelet Distribution Width, PCT–Plateletcrit

Supplementary Table 2 Results of the blood biochemistry test

Parameter	Conclusion	Result	Unit	Reference interval
**Glucose**	OK	4.93	mmol/L	4.11-7.95
**Urea**	OK	5.1	mmol/L	2.5-9.6
**Creatinine**	OK	57	µmol/L	44-159
**Urea/Creatinine**		22		
**Phosphorus**	OK	1.38	mmol/L	0.81-2.20
**Calcium**	OK	2.39	mmol/L	1.98-3.00
**Total Protein**	OK	56	g/L	52-82
**Albumin**	OK	26	g/L	23-40
**Globulin**	OK	30	g/L	25-45
**Albumin/Globulin**		0.9		
**ALT**	+	305	U/L	10-125
**ALP**	OK	131	U/L	23-212
**GGT**	OK	0	U/L	0-11
**Total bilirubin**	OK	<2	µmol/L	0-15
**Cholesterol**	OK	4.05	mmol/L	2.84-8.26
**Amylase**	OK	705	U/L	500-1500
**Lipase**	OK	643	U/L	200-1800
**Sodium**	-	140	mmol/L	144-160
**Chlorides**	-	108	mmol/L	109-122

ALT–Alanine Aminotransferase, ALP–Alkaline Phosphatase, GGT–Gamma-Glutamyltransferase

Supplementary Table 3 Serological examination for the detection of IgM and IgG antibodies

Viruses	Result	Interpretation
S–Canine Parvovirus (CPV) IgG No antibodies were detected in the serum sample (1:30).	N	negative
S-Canine Parvovirus (CPV) IgM Antibodies against Canine Parvovirus were detected in the serum sample at a titer of 1:90.	P	positive

Supplementary Table 4 Reference strains used in this study for the phylogenetic analysis

GenBank accession number	Variant	Strain / Isolate	Region	Collection year
EU659116	CPV-2	CPV-5.us.79	USA	1979
D26079	CPV-2a	Y1	Japan	1993
JQ686671	CPV-2a	CPV-2a	China	2011
JX660690	CPV-2a	SC02/2011	China	2011
KF149977	CPV-2a	2a_ME19_ECU2012	Ecuador	2012
KF366250	CPV-2a	CPV/915-H	India	2013
KR002802	CPV-2a	CPV/CN/SD9/2014	China	2014
KX434454	CPV-2a	CPV_IZSSI_29451_09	Italy	2009
LC214970	CPV-2a	CPV/dog/HCM/22/2013	Vietnam	2013
MF069443	CPV-2a	CPV/Raccoon/RC19/BC_2016	Canada	2016
MF177231	CPV-2a	260-00	Italy	2000
MF177265	CPV-2a	E12	Ecuador	2011
AY742934	CPV-2b	CPV-447	Germany	1995
EU659121	CPV-2b	CPV-411b .us.98	USA	1998
JN867606	CPV-2b	CPV-2b/Dog/US/19923/09	USA	2009
JQ268284	CPV-2b	CPV-LZ2	China	2011
KF149985	CPV-2b	2b_ME20_ECU2012	Ecuador	2012
KF482478	CPV-2b	1130	China	2009
KR002799	CPV-2b	CPV/CN/JL6/2013	China	2013
KR559895	CPV-2b	PT077/13	Portugal	2013
LC270891	CPV-2b	9985	Japan	2017
MH106699	CPV-2b	CPV-BJL2	China	2015
MH476588	CPV-2b	Canine/China/19/2017	China	2017
MT648206	CPV-2b	CPV-AHmas3	China	2018
OM721655	CPV-2b	CPV-2b-K5-TR	Turkey	2019
ON677437	CPV-2b	CPV-2b_IZSSI_2022PA2773	Italy	2022
ON733252	CPV-2b	FR1/CPV2-2021-HUN	Hungary	2021
OR463607	CPV-2b	IZSSI_2022PA15678idMeF	Italy	2022
FJ005204	CPV-2c	G333/99	Germany	1999
KM457120	CPV-2c	UY242	Uruguay	2010
KU508691	CPV-2c	HB	Australia	2015
KY073269	CPV-2c	UFMT	Brazil	2015
KY083093	CPV-2c	M26-6	Singapore	2014
LC216904	CPV-2c	CPV/dog/HCM/2/2013	Indonesia	2013
MF177227	CPV-2c	202-09	France	2009
MF177229	CPV-2c	368-12-17	Albania	2012
MF177242	CPV-2c	Arg26	Argentina	2008
MF510157	CPV-2c	CPV_IZSSI_2743_17	Italy	2017
MF805796	CPV-2c	Canine/China/08/2016	China	2016
MF918379	CPV-2c	GX-14	China	2018
MK144544	CPV-2c	K01708-1	South Korea	2017
MK517997	CPV-2c	CN/HB1709	China	2017
MN451682	CPV-2c	CPV609	Nigeria	2018
MN832850	CPV-2c	Taiwan/2018	Taiwan	2018
MT840294	CPV-2c	IZSSI_PA1464/19_idYV7_TR_4A72	Nigeria	2018
MW239578	CPV-2c	CPV4p-HN	Vietnam	2017
MW589468	CPV-2c	TRC-B90/TH/2020	Thailand	2020
MW659469	CPV-2c	157/2019	Romania	2019
OK094443	CPV-2	VNUA/CPV380-Hanoi	Vietnam	2020
OM100701	CPV-2c	EGY-FVMVL-36/2019	Egypt	2020

OM640098	CPV-2c	FM4	Canada	2018
OM937842	CPV-2c	16d	Ethiopia	2021
ON322838	CPV-2c	HN-100	China	2021
OP588002	CPV-2c	IZSVe_21/31130-1_dog_ITA	Italy	2021
OP611196	CPV-2c	Gab-9	Gabon	2019
OQ092740	CPV-2c	CPV/INDIA/AP45	India	2022
OQ198119	CPV-2c	ParvoviridaeDogfe435C1	China	2020/2021
OR399577	CPV-2c	CPV2/2022/3	China	2022/2023
OR463608	CPV-2c	IZSSI_2022PA17019idC1	Italy	2022
KX434462	FPLV	FPV_IZSSI_42807_15	Italy	2015

Abbreviations: CPV – canine parvovirus, FPVL – feline panleukopenia virus.

Supplementary Table 5. List of sequences producing significant alignments with the full-length VP2 gene sequence obtained in this study using the NCBI BLASTn algorithm (accessed June 21, 2024).

GenBank accession number	Pairwise identity [%]	Query coverage [%]	Variant	Origin	Year of collection
OP588003.1	99.83	100	CPV-2b	Italy	2021
ON733252.1	99.83	100	CPV-2b	Hungary	2021
ON185542.1	99.77	100	CPV-2b	Italy	2022
ON677437.1	99.77	100	CPV-2b	Italy	2022
ON322838.1	99.60	100	CPV-2c	China	2021
ON322808.1	99.60	100	CPV-2c	China	2020
OR463607.1	99.77	99	CPV-2c	Nigeria	2018
MN259001.1	99.54	100	CPV-2c	Nigeria	2018
MN258995.1	99.54	100	CPV-2c	China	2016
MN451682.1	99.54	100	CPV-2c	China	2017
MN451678.1	99.54	100	CPV-2c	China	2017
MK806279.1	99.54	100	CPV-2c	Thailand	2016
MF805796.1	99.54	100	CPV-2c	Thailand	2016
MG013488.1	99.54	100	CPV-2c	China	2017
MH711902.1	99.54	100	CPV-2c	China	2017
MH711894.1	99.54	100	CPV-2c	China	2017
MH476587.1	99.54	100	CPV-2c	China	2018
MH476585.1	99.54	100	CPV-2c	Italy	2017
MH476583.1	99.54	100	CPV-2c	Italy	2020
MF918379.1	99.54	100	CPV-2c	Italy	2022
MF510157.1	99.54	100	CPV-2c	Italy	2020
PP035147.1	99.54	100	CPV-2c	Italy	2021
PP035145.1	99.54	100	CPV-2c	Italy	2021
PP035144.1	99.54	100	CPV-2c	China	2022/2023
PP035141.1	99.54	100	CPV-2c	Indonesia	2013
PP035139.1	99.54	100	CPV-2c	China	2021
PP035138.1	99.54	100	CPV-2c	Gabon	2019
PP035136.1	99.54	100	CPV-2c	China	2020/2021
PP035134.1	99.54	100	CPV-2c	India	2022
PP035132.1	99.54	100	CPV-2c	Ethiopia	2021
PP035128.1	99.54	100	CPV-2c	China	2020
PP035127.1	99.54	100	CPV-2c	China	2021
PP035126.1	99.54	100	CPV-2c	China	2021
PP035125.1	99.54	100	CPV-2c	China	2021
PP035118.1	99.54	100	CPV-2c	China	2020
PP035116.1	99.54	100	CPV-2c	China	2020
PP035114.1	99.54	100	CPV-2c	China	2020

PP035112.1	99.54	100	CPV-2c	China	2020
OP588002.1	99.54	100	CPV-2c	China	2020
LC216904.1	99.54	100	CPV-2c	China	2020
OQ868530.1	99.54	100	CPV-2c	China	2020
OP611196.1	99.54	100	CPV-2c	China	2020
OQ198119.1	99.54	100	CPV-2c	China	2020
OQ092740.1	99.54	100	CPV-2c	China	2020
KP749851.1	99.54	100	CPV-2c	China	2020
KP715688.1	99.54	100	CPV-2c	China	2020
ON322847.1	99.54	100	CPV-2c	China	2020
ON322842.1	99.54	100	CPV-2c	China	2020
ON322839.1	99.54	100	CPV-2c	China	2020
ON322837.1	99.54	100	CPV-2c	China	2020
ON322836.1	99.54	100	CPV-2c	China	2020
ON322831.1	99.54	100	CPV-2c	China	2020
ON322826.1	99.54	100	CPV-2c	China	2020
ON322816.1	99.54	100	CPV-2c	China	2020
ON322814.1	99.54	100	CPV-2c	China	2020
ON322812.1	99.54	100	CPV-2c	China	2020
ON322811.1	99.54	100	CPV-2c	China	2020
ON322810.1	99.54	100	CPV-2c	China	2020
ON322807.1	99.54	100	CPV-2c	China	2020
ON322806.1	99.54	100	CPV-2c	China	2020
ON322803.1	99.54	100	CPV-2c	China	2019
ON322802.1	99.54	100	CPV-2c	China	2019
ON322798.1	99.54	100	CPV-2c	Canada	2018
ON322789.1	99.54	100	CPV-2c	China	2021
ON322787.1	99.54	100	CPV-2c	Vietnam	2020
ON322786.1	99.54	100	CPV-2c	Vietnam	2019
ON322785.1	99.54	100	CPV-2c	Vietnam	2018
ON322778.1	99.54	100	CPV-2c	Thailand	2020
ON322777.1	99.54	100	CPV-2c	Nigeria	2018
ON322761.1	99.54	100	CPV-2c	Nigeria	2018
ON322760.1	99.54	100	CPV-2c	Nigeria	2018
ON322759.1	99.54	100	CPV-2c	China	2019
ON322753.1	99.54	100	CPV-2c	China	2020
OK384308.1	99.54	100	CPV-2c	China	2020
MZ614964.1	99.54	100	CPV-2c	China	2019
OM640098.1	99.54	100	CPV-2c	China	2020
OM523076.1	99.54	100	CPV-2c	China	2019
OK094443.1	99.54	100	CPV-2c	China	2018
OK094440.1	99.54	100	CPV-2c	China	2019
OK094439.1	99.54	100	CPV-2c	China	2019
MW589468.1	99.54	100	CPV-2c	China	2019
MT840294.1	99.54	100	CPV-2c	Vietnam	2017
MT840293.1	99.54	100	CPV-2c	Korea	2017
MT648203.1	99.54	100	CPV-2c	Vietnam	2017
MW811189.1	99.54	100	CPV-2c	Vietnam	2017
MW650830.1	99.54	100	CPV-2c	China	2020
MZ506743.1	99.54	100	CPV-2c	China	2017
MT179773.1	99.54	100	CPV-2b	China	2018
MK144544.1	99.54	100	CPV-2c	Italy	2021
MT106236.1	99.54	100	CPV-2c	Italy	2022
MT106228.1	99.54	100	CPV-2c	Italy	2022
MK268683.1	99.49	100	CPV-2c	China	2022/2023
PP035148.1	99.49	100	CPV-2c	China	2022/2023
PP035140.1	99.49	100	CPV-2c	China	2022/2023
PP035131.1	99.49	100	CPV-2c	China	2022/2023
PP035124.1	99.49	100	CPV-2c	China	2022/2023

PP035123.1	99.49	100	CPV-2c	China	2022/2023
PP035122.1	99.49	100	CPV-2c	China	2020
PP049248.1	99.49	100	CPV-2c	China	2020
OR992670.1	99.49	100	CPV-2c	China	2021
